# Durability of response to icotrokinra for high‐impact site psoriasis: 1‐year ICONIC‐TOTAL findings

**DOI:** 10.1111/jdv.70471

**Published:** 2026-05-23

**Authors:** Richard B. Warren, Melinda Gooderham, Edward Lain, Robert Bissonnette, Yu‐Huei Huang, Julien Ringuet, Matthias Hoffmann, Andreas Pinter, Joseph F. Merola, Jessica H. Rubens, Dorothy Fan, Ming‐Chun Hsu, Shu Li, Ya‐Wen Yang, Cynthia M. C. DeKlotz, Eingun James Song

**Affiliations:** ^1^ Northern Care Alliance NHS Foundation Trust & Division of Musculoskeletal and Dermatological Sciences, Dermatology Centre, Manchester NIHR Biomedical Research Centre, Manchester Academic Health Science Centre University of Manchester Manchester UK; ^2^ SKiN Centre for Dermatology Peterborough Ontario Canada; ^3^ Department of Medicine Queen's University Kingston Ontario Canada; ^4^ Probity Medical Research Waterloo Ontario Canada; ^5^ Austin Institute for Clinical Research Sanova Dermatology Austin Texas USA; ^6^ Innovaderm Research Montreal Quebec Canada; ^7^ Department of Dermatology Chang Gung Memorial Hospital and College of Medicine, Chang Gung University Taoyuan City Taiwan; ^8^ Centre de Recherche Dermatologique du Québec Métropolitain Quebec Quebec Canada; ^9^ Department of Dermatology McGill University Montreal Quebec Canada; ^10^ Dermatology Practice Dr Matthias Hoffmann Witten Germany; ^11^ University Hospital Frankfurt am Main Frankfurt am Main Germany; ^12^ Department of Dermatology UT Southwestern Medical Center and O'Donnell School of Public Health Dallas Texas USA; ^13^ Division of Rheumatology, Department of Medicine UT Southwestern Medical Center and O'Donnell School of Public Health Dallas Texas USA; ^14^ Johnson & Johnson La Jolla California USA; ^15^ Johnson & Johnson Spring House Pennsylvania USA; ^16^ Johnson & Johnson West Chester Pennsylvania USA; ^17^ Johnson & Johnson Malvern Pennsylvania USA; ^18^ Johnson & Johnson Horsham Pennsylvania USA; ^19^ Frontier Dermatology Mill Creek Washington USA

**Keywords:** 1‐year, clinical trials, foot, genitalia, hand, high‐impact sites, icotrokinra, interleukin‐23, nails, oral, psoriasis, randomized controlled trials, scalp, skin, skin diseases

## Abstract

**Background:**

High‐impact site plaque psoriasis is difficult to treat. Icotrokinra, an oral peptide with high specificity for the interleukin (IL)‐23 receptor, demonstrated significantly higher rates of high‐impact site psoriasis clearance, versus placebo, with no safety signals, through Week (W)16.

**Objectives:**

Report clinical response rates and safety through 1 year of icotrokinra treatment in participants with high‐impact site plaque psoriasis.

**Methods:**

Participants (≥12 years of age; psoriasis body surface area ≥1%; Investigator's Global Assessment [IGA] ≥2) with at least moderate scalp, genital or hand/foot psoriasis were randomized (2:1) to once‐daily icotrokinra 200 mg (*N* = 208) or placebo (*N* = 103), with placebo‐to‐icotrokinra transition at W16 (*N* = 92). Rates (using nonresponder imputation) of achieving clear/almost clear (0/1) or clear (0) overall skin (IGA), genital (static Physician's Global Assessment of genitalia [sPGA‐G]), hand/foot (hf‐PGA) psoriasis and absent/very mild (0/1) or absent (0) scalp psoriasis (scalp‐specific‐IGA [ss‐IGA]), modified Nail Psoriasis Severity Index (mNAPSI) percent improvement and safety were assessed through W52.

**Results:**

Eighty‐eight per cent (275/311) of participants completed treatment through W52. In icotrokinra‐randomized participants, response rates increased through W24 and were durable through W52 for overall psoriasis clearance (IGA 0/1 range: 67%–70%) and across high‐impact sites (ss‐IGA 0/1: 72%–78%; sPGA‐G 0/1: 85%–90%; hf‐PGA 0/1: 54%–62%); responses were consistent among placebo‐randomized participants after transitioning to icotrokinra. High proportions of icotrokinra‐randomized participants achieved complete clearance during W24–52 (IGA 0: 44%–51%; ss‐IGA 0: 57%–66%; sPGA‐G 0: 73%–84%; hf‐PGA 0: 44%–58%). Mean mNAPSI improvement increased from W16 (33%) to W52 (62%). Exposure‐adjusted rates of participants with ≥1 adverse event (AE) or serious AE through W16 were similar between icotrokinra and placebo, with no increase in AE rates or occurrence of a safety signal through W52.

**Conclusions:**

Icotrokinra demonstrated high and durable rates of psoriasis clearance across high‐impact sites, with a favourable safety profile, through 1 year.

## Plain Language Summary

Psoriasis is a lifelong inflammatory skin disease causing thick, scaly plaques, which may be itchy or painful. Psoriasis can involve special body sites, such as the scalp, genitals, hands/feet and nails, which may be particularly bothersome to patients. Psoriasis is caused by high levels of certain immune proteins, including interleukin (IL)‐23. While many patients prefer oral treatments, current advanced oral therapies for psoriasis are less efficacious than injectable treatments and none specifically target the IL‐23 pathway.

Icotrokinra is an oral peptide that targets the IL‐23 receptor with high specificity to inhibit IL‐23 pathway signalling, and it is the first such molecule to gain approval for treatment of plaque psoriasis. Icotrokinra is being evaluated in the ICONIC‐TOTAL phase 3 randomized controlled trial conducted at 90 sites across 11 countries. Reported here are clinical response and safety through 1 year of icotrokinra treatment among 311 study participants with high‐impact site plaque psoriasis (scalp, genital and hand/foot). Previous reports showed icotrokinra had higher rates of psoriasis clearance compared with placebo through 16 weeks of treatment. After the placebo‐controlled period, among icotrokinra‐randomized participants, response rates increased through Week 24 and were durable through Week 52 for overall psoriasis clearance (ranging from 67%–70%) and across high‐impact sites (scalp: 72%–78%; genital: 85%–90%; hand/foot: 54%–62%). Additionally, icotrokinra provided improvements (62%) in nail psoriasis through Week 52. Icotrokinra safety through Week 16 was similar to placebo, with no increase in rates of adverse events through Week 52. Icotrokinra demonstrated high and durable rates of psoriasis clearance at high‐impact sites, with a favourable safety profile, through 1 year of treatment.

## Graphical abstract

Participants with at least moderate scalp, genital or hand/foot psoriasis receiving once‐daily oral icotrokinra achieved high, durable rates of overall and high‐impact site psoriasis clearance, showed substantial nail psoriasis improvement and exhibited no safety signals through 1 year, supporting icotrokinra use for long‐term management of high‐impact site psoriasis.

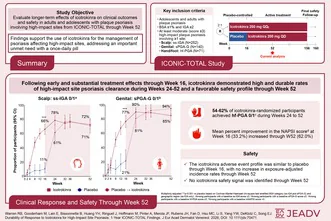

**Why was the study undertaken?**

- The phase 3 randomized, placebo‐controlled ICONIC‐TOTAL trial utilized a basket‐like design to concurrently assess 3 cohorts of participants with at least moderate scalp, genital or hand/foot psoriasis, including those with low body surface area psoriasis involvement, to evaluate the efficacy and safety of icotrokinra, an oral peptide with high specificity for the interleukin‐23 receptor and the first such molecule with this mechanism of action to gain approval for the treatment of plaque psoriasis.

**What does this study add?**

- Icotrokinra significantly improved skin, scalp and genital psoriasis. Response rates increased through W24 and were durable through W52, with 88%, 89% and 100% of those achieving ss‐IGA 0/1, sPGA‐G 0/1 and hf‐PGA 0/1, respectively, at W16 maintaining response at W52. The icotrokinra adverse event profile was similar to placebo through W16 and remained consistent through W52.

**What are the implications of this study for disease understanding and/or clinical care?**

- Icotrokinra has the potential to change the treatment landscape for patients with plaque psoriasis and psoriasis at high‐impact sites who prefer a safe and efficacious oral treatment option.

## INTRODUCTION

Psoriasis is a chronic, inflammatory skin disease affecting 1%–3% of the general population,^1^ with plaque psoriasis accounting for >80% of cases.^2^ Psoriasis can involve high‐impact sites, such as the scalp (~80% of psoriasis patients),^3^ genitals (~63%),^4^ hands/feet (12%–16%)^1^ and nails (80%–90%).^5^ While psoriasis at high‐impact sites may cover limited areas, the negative impact on health‐related quality of life (HRQoL) can be substantial.^1^, ^6^, ^7^ Additionally, these high‐impact regions can be difficult to treat.^8^ Nail improvement is particularly slow, requiring ~6–12 months for complete finger/toenail regrowth.^5^ As such, the International Psoriasis Council Delphi exercise recently classified patients with psoriasis involving special areas (e.g., face, palms, soles, genitalia and scalp) as candidates for systemic therapy,^9^ consistent with European and British guidelines that consider psoriasis location in treatment decisions.^10^, ^11^

Biologic therapies targeting cytokine pathways, including tumour necrosis factor (TNF), interleukin (IL)‐12/23, IL‐17 and IL‐23 signalling, are approved to treat moderate‐to‐severe psoriasis. While these therapies generally deliver greater efficacy than conventional treatments,^12^, ^13^ their injectable route of administration may pose a barrier for some patients, with many preferring oral treatment options due to convenience or needle phobia.^14^ In a survey of nearly 400 adults with psoriasis, oral therapy was the preferred route of administration.^15^ Both currently available advanced oral therapies, apremilast and deucravacitinib,^16^, ^17^ may have tolerability concerns and potentially lower efficacy compared with biologics, and neither is specifically approved for psoriasis in high‐impact sites.^18^ Thus, an unmet need remains for a targeted oral therapy, with efficacy comparable to biologics and a favourable safety profile, to treat patients with psoriasis at high‐impact body sites.

IL‐23 plays an important role in psoriasis immunopathogenesis, and inhibition of its pathway is clinically validated^19^ by therapies such as guselkumab,^20^ ustekinumab,^21^ tildrakizumab^22^ and risankizumab.^23^ In psoriasis, binding of IL‐23 to its receptor (IL‐23R) induces proinflammatory cytokine expression, among other inflammatory responses.^24^ Despite its critical role in psoriasis,^24^ the IL‐23R has not previously been a therapeutic target. Icotrokinra (JNJ‐77242113) exhibits high specificity for the IL‐23R to inhibit proximal signalling and downstream cytokine production, with no identified off‐target effects.^25^, ^26^, ^27^ Icotrokinra is approved for the treatment of moderate‐to‐severe plaque psoriasis in adults and paediatric patients 12 years of age and older who weigh at least 40 kg and are candidates for systemic therapy or phototherapy.^28^

Ongoing phase 3 studies evaluating icotrokinra, the first oral peptide targeting the IL‐23R to gain approval for treatment of plaque psoriasis, in participants with moderate‐to‐severe plaque psoriasis reported high rates of skin clearance.^29^, ^30^, ^31^ Icotrokinra demonstrated superior clinical responses in the ICONIC‐ADVANCE 1 and ICONIC‐ADVANCE 2 (NCT06143878; NCT06220604)^29^ studies (compared with placebo and deucravacitinib) and ICONIC‐LEAD (NCT06095115)^30^ study (compared with placebo), with no safety signals identified. The ICONIC‐TOTAL (NCT06095102) study was designed to assess clinical response and safety of icotrokinra among adults and adolescents with at least moderate plaque psoriasis affecting the scalp, genital or hand/foot regions and body surface area (BSA) ≥1%. As previously reported,^31^ significantly more icotrokinra‐treated than placebo‐treated participants achieved clear/almost clear overall, scalp and genital psoriasis at Week (W)16.^31^ Achievement rates of clear or almost clear hand/foot psoriasis were not significantly different between icotrokinra and placebo. Adverse event (AE) profiles were comparable between treatment groups through W16. Here, ICONIC‐TOTAL results through W52 are reported.

## METHODS

### Study design and participants

ICONIC‐TOTAL is an ongoing, phase 3, multicentre, randomized, double‐blind, placebo‐controlled, parallel‐group, interventional trial being conducted at 90 sites across 11 countries. The study design, eligibility criteria and protocol were previously reported.^31^ Eligible adults (≥18 years) and adolescents (≥12 to <18 years of age) had plaque psoriasis for ≥26 weeks at screening, total affected psoriasis BSA ≥1% and Investigator's Global Assessment (IGA) ≥2 and were candidates for phototherapy or systemic treatment and had inadequate response to ≥1 topical therapy. Participants had at least moderate psoriasis affecting high‐impact body sites, defined by a score ≥3 for one or more of the following: scalp‐specific IGA (ss‐IGA), static Physician's Global Assessment of Genitalia (sPGA‐G) and/or Physician's Global Assessment of hands and feet (hf‐PGA) score. ICONIC‐TOTAL included a placebo‐controlled period (W0–16), with participants randomized (2:1) to receive either once‐daily oral icotrokinra 200 mg or placebo, and an active‐treatment period (W16–156), when icotrokinra‐randomized participants continued their assigned treatment and placebo‐randomized participants started and continued icotrokinra. Results through W52 are reported here.

### Assessments

Study investigators graded the severity of overall skin psoriasis using IGA (range 0–4),^32^ scalp psoriasis using ss‐IGA (range 0–4)^33^ and Psoriasis Scalp Severity index (PSSI; range 0–72),^34^ genital psoriasis using sPGA‐G (range 0–5)^35^ and hand/foot psoriasis using hf‐PGA (range 0–4).^36^, ^37^, ^38^ Nail psoriasis severity was assessed via modified Nail Psoriasis Severity Index (mNAPSI; range 0–130; 0–13 for each fingernail).^39^

Participant‐reported assessments included overall itch using Psoriasis Symptom and Sign Diary (PSSD) Itch score (range 0–10), scalp itch using Scalp Itch numeric rating scale (NRS; range 0–10) and genital itch using Genital Psoriasis Symptom Scale (GPSS) NRS for itch (Item 1; range 0–10). Clinically meaningful improvements (CMIs) for all participant‐reported itch assessments were defined as ≥4‐point improvement from baseline.^40^, ^41^, ^42^ The proportion of participants achieving a PSSD symptom score of 0, among those with baseline PSSD >0, was also assessed.

Participants reported the impact of psoriasis and its treatment on HRQoL using the Dermatology Life Quality Index (DLQI; range 0–30)^43^ and Genital Psoriasis‐Sexual Frequency Questionnaire (GenPs‐SFQ Item 2; range 0–4).^44^

Reported AEs were coded using Medical Dictionary for Regulatory Activities (MedDRA) Version 27.0.

### Outcomes and analysis

ICONIC‐TOTAL sample size estimates, statistical considerations for imputation methods and multiplicity‐adjustment procedures applied in primary and key secondary endpoint analyses through W16 were previously reported.^31^ The primary and key secondary endpoints have been previously reported^31^; those reported here are outlined in the Data S1.

All participants continuing treatment after W16 received icotrokinra, and no additional formal hypothesis testing was planned. Clinical outcomes after W16 were analysed descriptively using summary statistics and 95% confidence intervals (CIs). The nonresponder imputation (NRI) method applied at W16 was used to handle missing data through W52 (see Supplemental Materials in Data S1).^31^

## RESULTS

### Participant disposition and baseline characteristics

**FIGURE 1:**
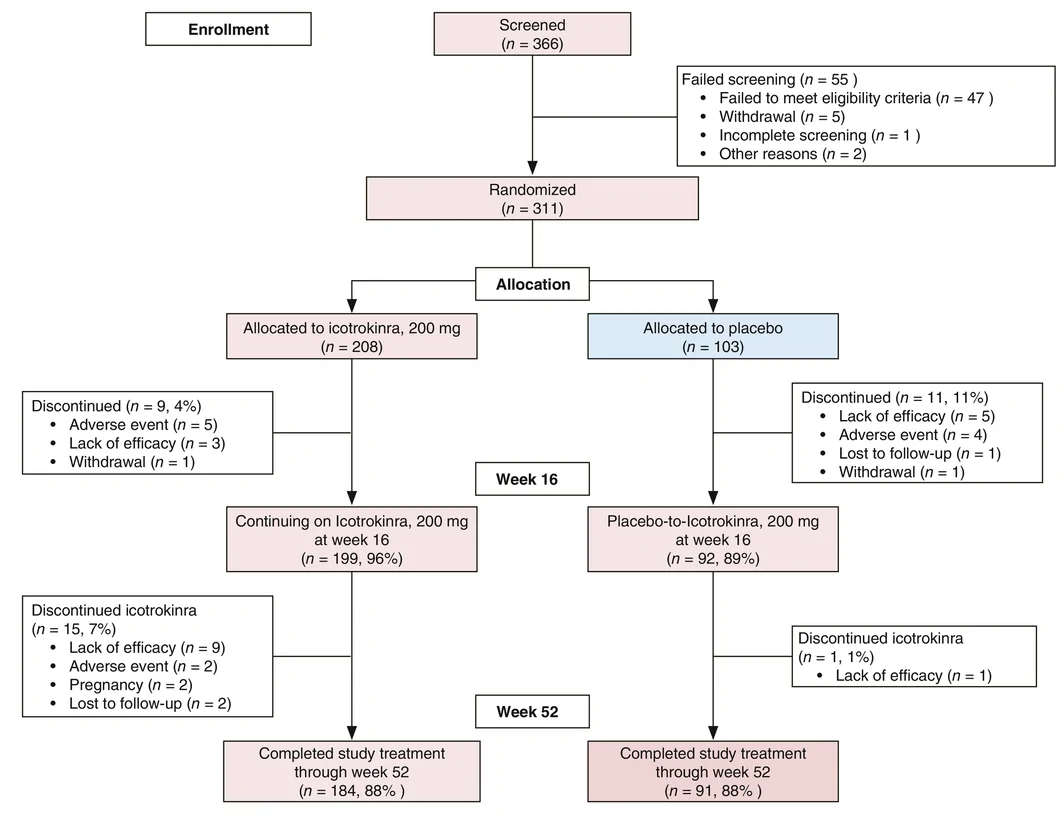
Participant disposition through Week 52 of the ICONIC‐TOTAL trial.

Among 311 randomized participants (208 icotrokinra, 103 placebo), 275 (88%) completed treatment through W52, including 184 (88%) icotrokinra‐randomized participants. Ninety‐two placebo‐randomized participants transitioned to icotrokinra at W16; of these 91 (99%) completed treatment through W52 (Figure 1).

Baseline characteristics were generally consistent between treatment groups.^31^ Briefly, 200 (64%) participants were male, 243 (78%) were white, mean (SD) age was 44.7 (14.3) years and 6 (2%) were adolescents (≥12 and <18 years of age). Overall, 252 (81%) participants had at least moderate scalp (ss‐IGA ≥3), 140 (45%) had genital (sPGA‐G ≥ 3) and 71 (23%) had hand/foot (hf‐PGA ≥3) involvement at baseline. Higher proportions of icotrokinra‐randomized participants with hand/foot psoriasis had severe disease at baseline (hf‐PGA = 4; 17 [35%]) compared with placebo‐randomized participants (4 [17%]). Among participants with mNAPSI score >0, the mean baseline score was higher among icotrokinra‐ (24.4) than placebo‐ (14.6) randomized participants.

### Response to icotrokinra through 1 year

#### Overall psoriasis, itch and HRQoL

**FIGURE 2:**
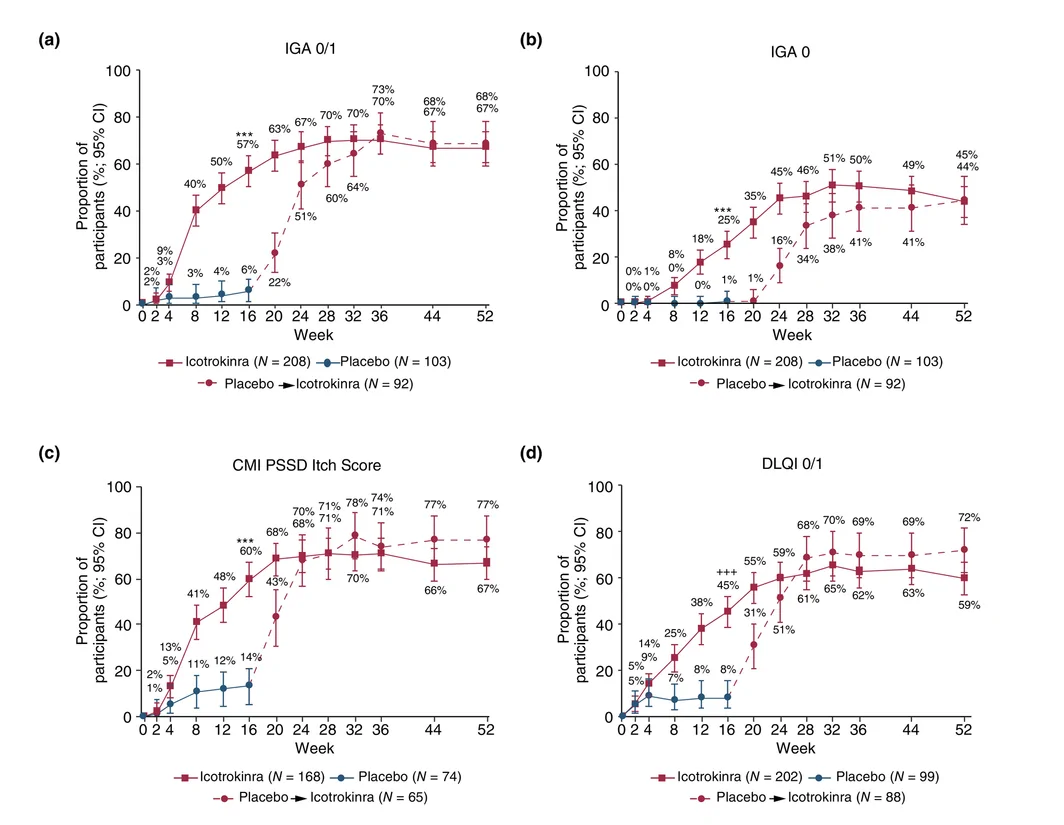
Proportion of participants achieving (a) overall IGA 0/1 with an improvement of ≥2 from baseline, (b) overall IGA 0, (c) CMI in PSSD Itch score and (d) DLQI 0/1 through Week 52. ***Multiplicity‐adjusted *p* < 0.001. ^+++^Nominal *p* < 0.001. PSSD itch evaluated among participants with baseline score ≥4. DLQI evaluated among participants with baseline score >1. IGA range: 0 (cleared); 1 (minimal); 2 (mild), 3 (moderate); 4 (severe). PSSD Itch range: 0–10; higher scores indicate worse severity. DLQI range: 0–1 (no effect); 2–5 (small effect); 6–10 (moderate effect); 11–20 (very large effect); 21–30 (extremely large effect). CI, confidence interval; CMI, clinically meaningful improvement; DLQI, dermatology life quality index; IGA, Investigator's Global Assessment; PSSD, psoriasis symptoms and signs diary.

As previously reported,^31^ the study's primary endpoint was met: 57% of icotrokinra‐randomized versus 6% of placebo‐randomized participants achieved IGA 0/1 response (IGA score of 0 or 1 with ≥2‐grade improvement from baseline) at W16 (multiplicity‐adjusted *p* < 0.001). IGA 0/1 response rates increased over time and remained high from W24 to W52 among icotrokinra‐randomized participants (ranging from 67%–70%; Figure 2a). Among 118 icotrokinra‐randomized participants achieving IGA 0/1 at W16, 87% maintained this response at W52. A significantly higher proportion of icotrokinra‐ versus placebo‐randomized participants achieved complete skin clearance (IGA 0) at W16 (25% vs. 1%; multiplicity‐adjusted *p* < 0.001). Among icotrokinra‐randomized participants, IGA 0 response rates increased through W24 (45%) and were consistent through W52 (44%; Figure 2b). After transition to icotrokinra, the placebo‐to‐icotrokinra group achieved comparable IGA 0/1 and IGA 0 response rates (Figure 2a,b).

Among 168 icotrokinra‐randomized participants with baseline PSSD Itch score ≥4, the rate of achieving CMI in PSSD Itch at W16 (60%) increased over time and remained high from W24 to W52 (66%–71%; Figure 2c). Among 191 icotrokinra‐randomized participants with baseline PSSD symptom score >0, 38% achieved PSSD 0 by W52. Among 202 icotrokinra‐randomized participants with baseline DLQI >1, the proportion reporting DLQI 0/1 (no impact of psoriasis on HRQoL) increased through W24 (59%) and was consistent through W52 (59%; Figure 2c). Placebo‐randomized participants reported comparable improvements in symptoms and HRQoL after transitioning to icotrokinra (Figure 2c,d).

#### Scalp psoriasis

**FIGURE 3:**
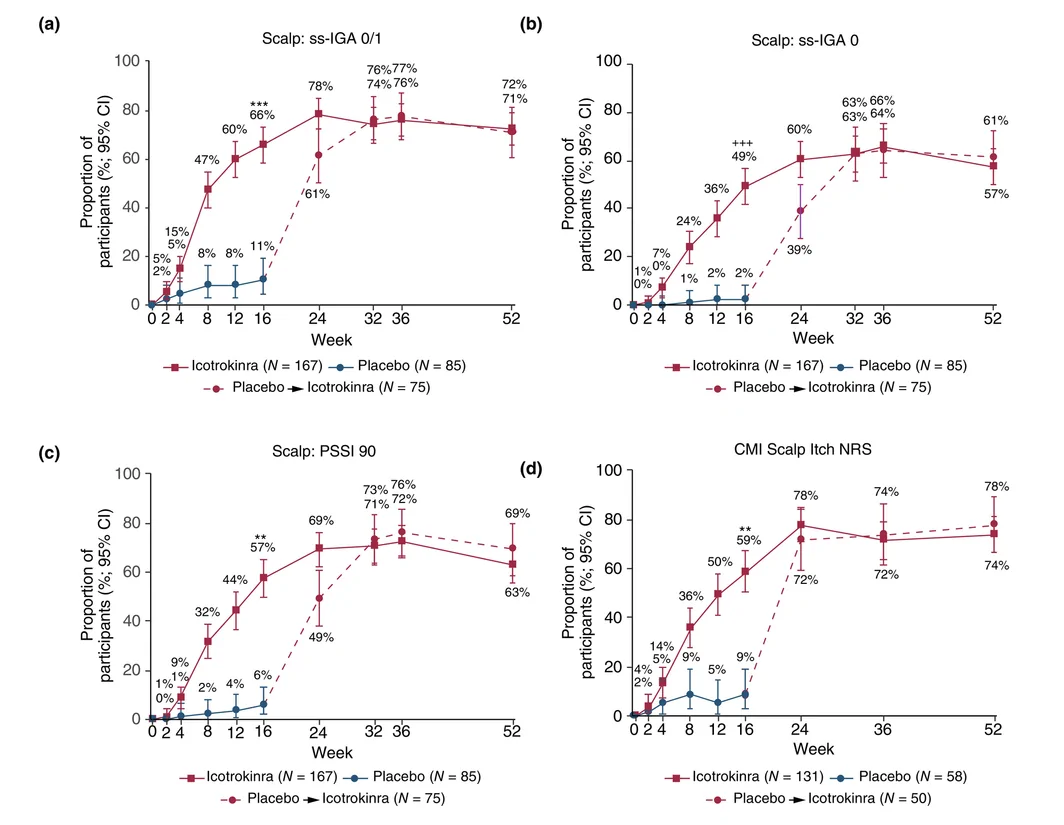
Proportion of participants achieving the following improvements in scalp psoriasis: (a) ss‐IGA 0/1, (b) ss‐IGA 0, (c) PSSI 90 and (d) CMI in Scalp Itch NRS through Week 52. ***Multiplicity‐adjusted *p* < 0.001. **Multiplicity‐adjusted *p* < 0.01. ^+++^Nominal *p* < 0.001. ss‐IGA and PSSI evaluated among participants with baseline ss‐IGA score ≥3. Scalp Itch NRS evaluated among participants with baseline score ≥4 and ss‐IGA score ≥3. ss‐IGA range: 0 (absent), 1 (very mild), 2 (mild), 3 (moderate), 4 (severe). PSSI range: 0–72; higher scores indicate worse severity. Scalp Itch NRS range: 0–10; higher scores indicate worse scalp itch. CI, confidence interval; CMI, clinically meaningful improvement; NRS, numeric rating scale; PSSI, psoriasis scalp severity index; ss‐IGA, scalp specific‐Investigator's Global Assessment.

Among participants with at least moderate scalp psoriasis (ss‐IGA score ≥3), icotrokinra demonstrated a significantly higher ss‐IGA 0/1 response rate (66% of 167) versus placebo (11% of 85) at W16 (multiplicity‐adjusted *p* < 0.001).^31^ The rate of achieving absent/very mild scalp psoriasis among icotrokinra‐randomized participants increased through W24 (78%) and was consistent through W52 (72%; Figure 3a). Among 110 icotrokinra‐randomized participants achieving ss‐IGA 0/1 at W16, 88% maintained this response through W52. The proportion of icotrokinra‐randomized participants achieving ss‐IGA 0 also increased through W24 (60%) and was consistent at W52 (57%; Figure 3b). PSSI 90 response rates in icotrokinra‐randomized participants were consistent with ss‐IGA, increasing through W24 and remaining high through W52 (63%–72%; Figure 3c). Placebo‐randomized participants demonstrated comparable improvements in scalp psoriasis after transitioning to icotrokinra (Figure 3a–c).

Among 131 icotrokinra‐randomized participants with baseline Scalp Itch NRS ≥4 and ss‐IGA score ≥3, the proportion of participants reporting CMI in Scalp Itch NRS at W16 (59%) increased through W24 (78%) and remained high through W52 (74%; Figure 3d). Placebo‐randomized participants who transitioned to icotrokinra reported comparable rates of CMI in Scalp Itch NRS (Figure 3d).

#### Genital psoriasis

**FIGURE 4:**
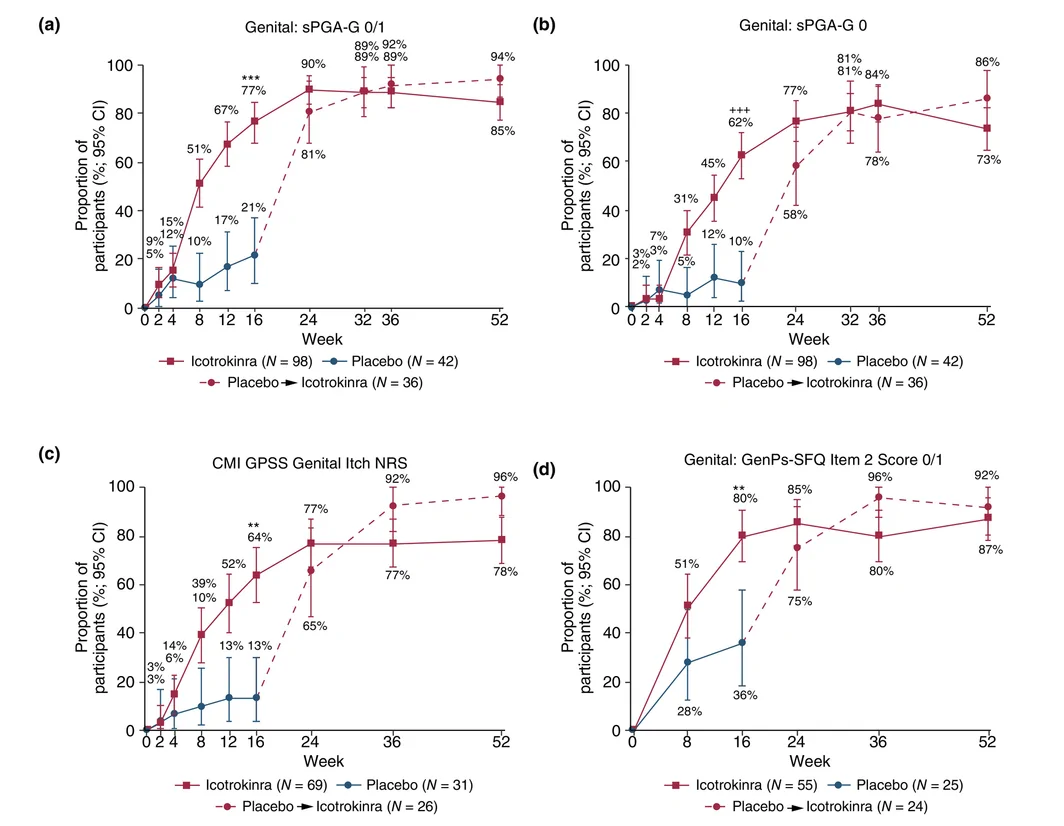
Proportion of participants achieving the following improvements in genital psoriasis: (a) sPGA‐G 0/1, (b) sPGA‐G 0, (c) CMI in GPSS Genital Itch NRS and (d) GenPs‐SFQ Item 2 score 0/1 through Week 52. ***Multiplicity‐adjusted *p* < 0.001. **Multiplicty‐adjusted *p* < 0.01. ^+++^Nominal *p* < 0.001. sPGA‐G evaluated among participants with baseline score ≥3. GPSS Genital Itch NRS evaluated among participants with baseline score ≥4 and sPGA‐G ≥3. sPGA‐G range: 0 (clear), 1 (minimal), 2 (mild), 3 (moderate), 4 (severe), 5 (very severe). GPSS Genital Itch NRS range: 0–10; higher scores indicate worse genital itch. GenPs‐SFQ Item 2 (In the past week, how often did your genital psoriasis limit the frequency of your sexual activity?) range: 0 (never), 1 (rarely), 2 (sometimes), 3 (often), 4 (always). CI, confidence interval; CMI, clinically meaningful improvement; GenPs‐SFQ, genital psoriasis sexual frequency questionnaire; GPSS, genital psoriasis symptoms scale; NRS, numeric rating scale; sPGA‐G, static Physician's Global Assessment of Genitalia.

Among participants with at least moderate genital psoriasis (sPGA‐G score ≥3), 77% of 98 icotrokinra‐randomized versus 21% of 42 placebo‐randomized participants achieved clear/minimal genital psoriasis (sPGA‐G 0/1) at W16 (multiplicity‐adjusted *p* < 0.001).^31^ Rates of achieving clear/minimal genital psoriasis among icotrokinra‐randomized participants increased through W24 (90%) and were consistent through W52 (85%; Figure 4a). Among 75 icotrokinra‐randomized participants who achieved sPGA‐G 0/1 at W16, 89% maintained this response at W52. The majority of icotrokinra‐randomized participants achieved completely clear genital psoriasis (sPGA‐G 0) from W24 through W52 (73%–84%; Figure 4b). Placebo‐randomized participants achieved comparable improvements in genital psoriasis after transitioning to icotrokinra (Figure 4a,b).

Among participants with baseline GPSS Genital Itch NRS ≥4 and sPGA‐G ≥3, 64% of 69 receiving icotrokinra versus 13% of 31 receiving placebo reported CMI in GPSS Genital Itch NRS at W16 (multiplicity‐adjusted *p* = 0.008).^31^ Rates of CMI in GPSS Genital Itch NRS among icotrokinra‐randomized participants increased through W24 (77%) and remained high through W52 (78%), with comparable response rates among placebo‐randomized participants after transition to icotrokinra (Figure 4c).

Among participants with baseline GenPs‐SFQ Item 2 score ≥2 and sPGA‐G score ≥3, 80% of 55 icotrokinra‐randomized versus 36% of 25 placebo‐randomized participants reported psoriasis symptoms never/rarely impacted frequency of sexual activity (GenPs‐SFQ Item 2 score of 0/1) at W16 (multiplicity‐adjusted *p* = 0.008).^31^ From W24 through W52, 85%–87% of icotrokinra‐randomized participants reported GenPs‐SFQ Item 2 0/1 response (Figure 4d). High proportions of icotrokinra‐randomized participants reported no impact of psoriasis symptoms on sexual frequency (GenPs‐SFQ Item 2 score of 0) from W24 through W52 (71%–73%; Figure S1). Placebo‐randomized participants reported comparable improvements in psoriasis impact on sexual frequency after transitioning to icotrokinra (Figure 4d, Figure S1).

#### Hand/foot psoriasis

Among participants with at least moderate hand/foot psoriasis (hf‐PGA ≥3), 42% (95% CI: 28%–56%) of 48 icotrokinra‐randomized versus 26% (95% CI: 10%–48%) of 23 placebo‐randomized participants achieved clear or almost clear hand/foot psoriasis (hf‐PGA 0/1) at W16 (multiplicity‐adjusted *p* = 0.144).^31^ Rates of hf‐PGA 0/1 achievement increased among icotrokinra‐randomized participants through W24 (54%) and were consistent through W52 (62%; Figure S2A). Among the 20 icotrokinra‐randomized participants achieving hf‐PGA 0/1 at W16, 100% maintained this response at W52. At W52, 58% of icotrokinra‐randomized participants exhibited clear hand/foot psoriasis (hf‐PGA 0), with comparable findings following placebo‐to‐icotrokinra transition (Figure S2B).

#### Nail psoriasis

Among 85 icotrokinra‐randomized participants with baseline mNAPSI score >0, mean percent improvement in the NAPSI score at W16 (33.2%) increased through W52 (62.0%; Figure S2C). More than one‐third of icotrokinra‐treated participants achieved completely clear nail psoriasis (mNAPSI 0) at W52.

### Safety

Safety findings through W16 have been reported.^31^ Through W52, 300 participants received ≥1 dose of icotrokinra, including 92 participants who transitioned from placebo to icotrokinra at W16. The mean duration of follow‐up was 45.3 weeks (Table 1).

**TABLE 1:** Summary of adverse events through Week 52.

	Placebo‐controlled (W0‐16)		W16–52	Through W52	
	Placebo	Icotrokinra	Placebo‐to‐Icotrokinra	Icotrokinra	Icotrokinra combined
	(*N* = 103)	(*N* = 208)	(*N* = 92)	(*N* = 208)	(*N* = 300)
Mean weeks of follow‐up	15.6	16.0	36.2	49.3	45.3
Participant‐years of follow‐up	30.8	63.6	63.9	196.4	260.2
No. (%) of participants with ≥1 AE	46 (44.7%)	105 (50.5%)	51 (55.4%)	153 (73.6%)	204 (68.0%)
Participants/100 PYs (95% CI)^a^	217.0 (154.3, 279.7)	232.8 (188.2, 277.3)	132.0 (95.8, 168.3)	168.5 (141.8, 195.2)	157.6 (136.0, 179.3)
No. (%) of participants with ≥1 SAE	2 (1.9%)	1 (0.5%)	1 (1.1%)	6 (2.9%)	7 (2.3%)
Participants/100 PYs (95% CI)^a^	6.6 (0, 15.7)	1.6 (0, 4.7)	1.6 (0, 4.7)	3.1 (0.6, 5.6)	2.7 (0.7, 4.8)
No. (%) of participants with an AE leading to discontinuation	4 (3.9%)	6 (2.9%)	0 (0.0%)	7 (3.4%)	7 (2.3%)
Participants/100 PYs (95% CI)^b^	13.2 (3.6, 33.7)	9.6 (3.5, 20.8)	0 (0, 4.7)	3.6 (1.4, 7.4)	2.7 (1.1, 5.6)
No (%) of participants with ≥1 infection	23 (22.3%)	59 (28.4%)	39 (42.4%)	106 (51.0%)	145 (48.3%)
Participants/100 PYs (95% CI)^a^	88.3 (52.2, 124.4)	110.0 (81.9, 138.1)	80.9 (55.5, 106.3)	81.1 (65.6, 96.5)	81.0 (67.8, 94.2)
No. (%) of participants with ≥1 serious infection	1 (1.0%)	0 (0%)	0 (0%)	0 (0%)	0 (0%)
Participants/100 PYs (95% CI)^b^	3.3 (0.1, 18.1)	0 (0, 4.7)	0 (0, 4.7)	0 (0, 1.5)	0 (0, 1.2)
No. (%) of participants with ≥1 gastrointestinal AE	8 (7.8%)	15 (7.2%)	7 (7.6%)	21 (10.1%)	28 (9.3%)
Participants/100 PYs (95% CI)^a^	27.0 (8.3, 45.6)	24.8 (12.3, 37.4)	11.5 (3.0, 20.0)	11.6 (6.6, 16.6)	11.6 (7.3, 15.9)
No. (%) of participants with ≥1 malignancy^c^	0 (0%)	1 (0.5%)	0 (0%)	2 (1.0%)	2 (0.7%)
Participants/100 PYs (95% CI)^b^	0 (0, 9.7)	1.6 (0, 8.8)	0 (0, 4.7)	1.0 (0.1, 3.7)	0.8 (0.1, 2.8)

*Note*: Data presented is *n* (%), unless otherwise noted. Placebo‐to‐icotrokinra column only includes data after Week 16 for placebo participants who transitioned to icotrokinra. Combined column includes all participants who received ≥1 dose of icotrokinra. The most common AEs (≥5% of participants in any group) through W16 were nasopharyngitis (icotrokinra: 12%; placebo: 11%), upper respiratory tract infection (icotrokinra: 4%; placebo: 5%) and headache (icotrokinra: 3%; placebo: 6%). The most common AEs (≥5% of participants in any group) through W52 were nasopharyngitis (icotrokinra: 21%; placebo‐to‐icotrokinra: 17%) and upper respiratory tract infection (icotrokinra: 11%; placebo‐to‐icotrokinra: 10%).

Abbreviations: AE, adverse event; CI, confidence interval; No, number; PY, patient‐years; SAE, serious adverse event; W, week.

^a^ Confidence intervals were based on a Wald statistic using the normal assumption.

^b^ Confidence intervals were based on an exact method assuming that the observed number of events follows a Poisson distribution.

^c^ Includes chronic lymphocytic leukaemia and malignant melanoma in situ.

Through W16, the most common AEs (≥5% of participants in any group) were nasopharyngitis (icotrokinra: 12%; placebo: 11%), upper respiratory tract infection (icotrokinra: 4%; placebo: 5%) and headache (icotrokinra: 3%; placebo: 6%). These AEs remained the most commonly occurring events with continued icotrokinra treatment through W52 (Table 1). Exposure‐adjusted incidence rates of icotrokinra‐randomized participants with ≥1 AE did not increase between the W0–16 (232.8 [188.2, 277.3]/100PY) and W0–52 (168.5 [141.8, 195.2]/100 PY) time periods. Findings were similar for icotrokinra‐randomized participants with ≥1 SAE, AE leading to discontinuation, infection, serious infection, gastrointestinal AE or malignancy (Table 1). Among placebo‐randomized participants who transitioned to icotrokinra at W16, the exposure‐adjusted rates of participants with ≥1 AE did not increase through W52 for AEs overall (placebo W0–16: 217.0 [154.3, 279.7]; placebo‐to‐icotrokinra W16–52: 132.0 [95.8, 168.3]/100 PY) or across specific AE categories (Table 1).

Through W16, a single event of malignancy (melanoma; assessed to be unrelated to the study drug by the investigator) was observed in an icotrokinra‐treated participant with a history of melanoma.^31^ During W16–W52, a second malignancy event (chronic lymphocytic leukaemia) was reported in a participant with a history of neutropenia (see Supplementary materials in Data S1).

## DISCUSSION

In the phase 3 ICONIC‐TOTAL study of adults and adolescents with at least moderate scalp, genital or hand/foot psoriasis, rates of psoriasis improvement and clearance increased over time and remained high through 1 year of treatment, with nearly half of icotrokinra‐treated participants achieving complete overall skin clearance. Approximately 60% of affected icotrokinra‐randomized participants demonstrated completely clear scalp and hand/foot psoriasis at 1 year, and ~70% exhibited complete clearance of genital psoriasis. Similar improvements in high‐impact site psoriasis were reported among placebo‐randomized participants who transitioned to icotrokinra treatment after W16. Meaningful improvements in overall, scalp and genital itch were reported, with increasing proportions of icotrokinra‐randomized participants reporting reduced itch through W24 and durable response rates through W52. These clinical benefits translated to improved quality of life, with ~60% of participants reporting no impact of psoriasis on overall HRQoL from W24 through W52 of treatment.

These notable rates of high‐impact site psoriasis clearance are important given the difficulty treating these areas and how disproportionately they impact patients' HRQoL, even when the overall BSA with psoriasis involvement is low.^45^ Genital psoriasis has a significant impact on intimacy and psychological well‐being^46^ and is frequently undertreated due to reluctance to discuss sensitive issues.^47^ Icotrokinra's effectiveness in reducing genital psoriasis symptoms and impact on frequency of sexual activity suggest a high therapeutic value for these patients.

Nail psoriasis is particularly difficult to treat.^48^ Despite more severe nail psoriasis at baseline in the icotrokinra vs. placebo‐to‐icotrokinra group, icotrokinra provided comparable improvements in nail psoriasis by W52 (mNAPSI mean improvement: 62% and 59%, respectively). About one‐third of participants with nail psoriasis at baseline reported complete resolution of nail psoriasis by W52.

While the difference in proportions of icotrokinra‐ and placebo‐randomized participants achieving clear or almost clear hand/foot psoriasis (hf‐PGA 0/1) at W16 (42% vs. 26%) did not reach statistical significance,^31^ the treatment effect at this earlier timepoint may have been impacted by the higher prevalence of severe disease (hf‐PGA = 4) among icotrokinra‐randomized participants at baseline, as well as the limited number of participants (*N* = 71) in this cohort. Nonetheless, across groups, 62%–68% of icotrokinra‐treated participants achieved hf‐PGA 0/1 and nearly 60% achieved hf‐PGA 0 by W52.

W52 results from the ICONIC‐TOTAL study complement findings from placebo‐controlled periods of the ongoing ICONIC‐LEAD (NCT06095115)^30^ and ICONIC‐ADVANCE 1 and 2 (NCT06143878; NCT06220604)^29^ studies in participants with moderate‐to‐severe plaque psoriasis (BSA ≥10%, PASI ≥12, IGA ≥3), which found over 60% of icotrokinra‐treated versus ~10% of placebo‐treated participants achieved IGA 0/1 at W16 (*p* < 0.001 in each study). The present findings are also supported by results from ICONIC‐LEAD participants with at least moderate high‐impact site psoriasis: at W24 high proportions of icotrokinra‐randomized participants achieved ss‐IGA 0/1 (81%) and ss‐IGA 0 (65%); sPGA‐G 0/1 (80%) and sPGA‐G 0 (70%); and hf‐PGA 0/1 (76%) and hf‐PGA 0 (62%), with high percent improvements in mNAPSI as early as W16 (44%).^49^ Thus, icotrokinra has demonstrated robust and durable improvements in difficult‐to‐treat, high‐impact site plaque psoriasis in a broad range of participants with psoriasis.

No safety signals were observed through 1 year of icotrokinra exposure in ICONIC‐TOTAL. AE rates were similar between icotrokinra‐ and placebo‐randomized participants through W16, with no increases in exposure‐adjusted rates in icotrokinra‐randomized participants through 1 year of treatment. Similarly, exposure‐adjusted rates of participants with ≥1 AE did not increase among placebo‐randomized participants after transition to icotrokinra at W16. These findings are consistent with the 1‐year icotrokinra safety profile reported from the phase 2 FRONTIER studies,^50^, ^51^ through 6 months^30^ and 1 year^52^ of the ongoing ICONIC‐LEAD study, and through W24 of the ICONIC‐ADVANCE 1 and 2 studies.^29^ The ICONIC‐ADVANCE 1 and 2 studies reported a safety profile comparable to placebo, with lower AE and infection rates compared to deucravacitinib.^29^ Future analyses comparing icotrokinra malignancy rates with the national cancer statistics from the United States National Institute of Health Surveillance, Epidemiology, and End Results Program (SEER) database will further contextualize safety findings.

Injectable biologics targeting the IL‐23 pathway are safe and effective in treating psoriasis in high‐impact areas;^33^, ^53^, ^54^ however, patients and healthcare providers often prefer oral administration.^15^ Oral treatments with efficacy comparable to injectables are currently lacking from the treatment landscape. Noting the inherent limitations of direct cross‐study comparisons, response rates observed with icotrokinra in treating high‐impact site psoriasis were within the range of those reported for biologics targeting IL‐23^33^, ^55^ or IL‐17.^56^, ^57^ Recently reported findings from comparative network meta‐analyses (NMAs) of icotrokinra and approved advanced psoriasis treatments in patients with moderate‐to‐severe plaque psoriasis indicate icotrokinra is comparable to IL‐23/17 inhibitor injectables and superior to advanced oral therapies in achieving completely clear skin (in both PASI 100 and IGA 0 NMAs) at W16.^58^ Future indirect treatment comparison analyses will be important to further inform treatment decisions.

ICONIC‐TOTAL employed validated measures to assess the effect of icotrokinra. Stringent methods of data analysis, including nonresponder imputation, were used to estimate response rates. High participant retention and the inclusion of both adolescent and adult participants with a broad range of affected BSA enabled a comprehensive, longer‐term assessment of icotrokinra response in a diverse psoriasis population. This approach addresses a recognized gap in the psoriasis literature, with most prior studies focusing on adults with high BSA involvement.^59^ Continued follow‐up in this 3‐year study will provide the opportunity to fully characterize the durability of icotrokinra effects on high‐impact site plaque psoriasis. This study was limited by the small sample size per high‐impact site, particularly among participants with hand/foot psoriasis; however, ongoing phase 3 icotrokinra studies may address these limitations. The larger ICONIC‐LEAD^30^ and ICONIC‐ADVANCE 1 and 2 studies^29^ include participants with high‐impact site psoriasis treated with icotrokinra, allowing for pooled analyses with larger sample sizes and longer follow‐up periods. Finally, all placebo‐randomized participants transitioned to icotrokinra treatment at W16. As such, all observations after W16 are descriptive in nature.

## CONCLUSIONS

Icotrokinra, an oral peptide with high specificity for the IL‐23R to inhibit IL‐23 pathway signalling, is the first molecule with this mechanism of action to gain approval for treatment of plaque psoriasis. In the current study, icotrokinra demonstrated high rates of overall skin, scalp, genital and hand/foot psoriasis clearance, and improvements in nail psoriasis, through 1 year in adults and adolescents with at least moderate high‐impact area plaque psoriasis. The icotrokinra AE profile, which was similar to placebo, remained favourable and no safety signals were identified through 1 year.

## AUTHOR CONTRIBUTIONS

Study design: R.B.W, M.G., E.L., R.B., J.H.R, M.‐C.H., S.L., C.M.C.D. Gathered data: R.B.W., M.G., E.L., R.N., Y.‐H.H., J.R., M.H., A.P., J.F.M., J.H.R., D.F., S.L., Y.‐W.Y., C.M.C.D., E.J.S. All authors contributed to data analysis and collaborated on writing the manuscript.

## FUNDING INFORMATION

This research was funded by Johnson & Johnson and supported by the National Institute for Health and Care Research (NIHR) Manchester Biomedical Research Centre (BRC) (NIHR203308).

## CONFLICT OF INTEREST STATEMENT

**RBW**: Consultant and/or speaker for AbbVie, Almirall, Alumis, Amgen, Apogee, Arena, Astellas, Avillion, Biogen, Boehringer Ingelheim, Bristol‐Myers Squibb, Celgene, DICE Therapeutics, Eli Lilly, Galderma, GSK, Johnson & Johnson, LEO Pharma, Meiji Pharma, Nektar Therapeutics, Novartis, Pfizer, RAPT Pharmaceuticals, Sanofi, Sitryx, Sun Pharma, UCB and UNION; Received research grants from AbbVie, Almirall, Amgen, Celgene, Eli Lilly, Johnson & Johnson, LEO Pharma, Novartis, Pfizer and UCB. **MG**: Speaker, investigator or advisory board member for AbbVie, Acelyrin, Alumis, Amgen, Akros, AnaptysBio, Apogee, Arcutis, Arena, Aslan, Bausch Health, Boehringer Ingelheim, Bristol‐Myers Squibb, Coherus, Dermira, Dermavant, Eli Lilly, Galderma, GSK, Incyte, Inmagene, Johnson & Johnson, JAMP Pharma, Kyowa Kirin, LEO Pharma, Medimmune, Merck, Moonlake, Nimbus, Novartis, Pfizer, Regeneron, Reistone, Sanofi, Genzyme, Sun Pharma, Tarsus, Takeda, Union, UCB and Vyne. **EL**: Speaker, consultant, advisory board member or principal investigator for AbbVie, Aslan Pharmaceuticals, Biofrontera, Caliway, Cassiopea, Castle Biosciences, Concert Pharmaceuticals, Celldex Therapeutics, Eli Lilly, Evoimmune, Galderma, Incyte, Johnson & Johnson, L'Oréal, Nielsen BioSciences, Peregrine Ophtalmic, Pfizer, Regeneron, RegenX Science, Sanofi, Symatese, Takeda, Teoxane, UCB, Veradermics and Vyne. **RB**: Advisory board member, consultant, speaker and/or investigator for and received honoraria and/or grants from AbbVie, Alumis, Amgen, AnaptysBio, Arcutis, Bristol‐Myers Squibb, Celgene, Dermavant, Eli Lilly, Johnson & Johnson, LEO Pharma, Nimbus, Takeda, UCB, VentyxBio, Vyne, Xencor, Zai Lab and Zurabio; Employee and shareholder of Innovaderm Research. **Y‐HH**: Conducted clinical trials for or received honoraria as a consultant for AbbVie, Bristol‐Myers Squibb, Celgene, Johnson & Johnson, Novartis and Pfizer. **JR**: Consultant and/or speaker for AbbVie, Amgen, Apogee, Arcutis, Bausch Health, Boehringer Ingelheim, Bristol‐Myers Squibb, Eli Lilly, Galderma, Incyte, Johnson & Johnson, JAMP Pharma, LEO Pharma, L'Oréal, NKS Health, Novartis, Organon, Pfizer, Sandoz, Sanofi, Genzyme, Sun Pharma, UCB. Participated as an Investigator in clinical trials for AbbVie, Amgen, Apogee, Alumis, Aristea, ASLAN Therapeutics, Bristol‐Myers Squibb, Celgene, Concert Pharmaceuticals, Correvitas, DICE Therapeutics, Eli Lilly, Incyte, Innovaderm, Johnson & Johnson, Kyowa Kirin, LEO Pharma, Merck, Novartis, Pfizer, Sanofi, Genzyme, Sun Pharma and UCB. **MH**: Advisory board member, consultant, speaker and/or investigator for and/or received honoraria and/or grants from AbbVie, Almirall, Biofrontera, Bristol‐Myers Squibb, Dermapharm, Dermasence, Eli Lilly, Forward, Galderma, Incyte, Johnson & Johnson, Kiniksa, LEO Pharma, L'Oréal, MSD, Nektar Therapeutics, Novartis, Pierre Fabre, Pfizer, Sanofi, Schering‐Plough, Smerud Medical Research, Stallergenes Greer, Viatris and UCB. **AP**: Advisor and/or received speaker's honoraria and/or received grants and/or participated in clinical trials for AbbVie, Almirall, Amgen, Biogen, BioNTech, Boehringer Ingelheim, Celgene, Celltrion, Eli Lilly, Galderma, GSK, Hexal, Johnson & Johnson, Klinge Pharma, LEO Pharma, MC2, Medac, Merch Serono, Mitsubishi, MSD, Novartis, Pascoe, Pfizer, Regeneron, Roche, Sandoz, Sanofi, Genzyme, Schering‐Plough, Tigercat Pharma, UCB and Zuellig Pharma. **JFM**: Consultant and/or investigator for AbbVie, Amgen, Astrazeneca, Biogen, Boehringer Ingelheim, Bristol‐Myers Squibb, Dermavant, Eli Lilly, Galderma, Johnson & Johnson, Moonlake, Novartis, Oruka, Pfizer, Regeneron, Sanofi, Sun Pharma and UCB. **JHR, DF, M‐CH, SL, Y‐WY and CMCD**: Employees of Johnson & Johnson may own stock/stock options in Johnson & Johnson. **EJS**: Investigator, consultant and/or speaker for AbbVie, Acelyrin, Alphyn, Alumis, Amgen, Apogee, Arcutis, ASLAN, Boehringer Ingelheim, Bristol‐Myers Squibb, Celldex, CorEvitas, Dermavant, DermBiont, Eli Lilly, Galderma, Incyte, Johnson & Johnson, LEO Pharma, MoonLake, Novartis, Pfizer, Regeneron, Sanofi, Genzyme, Sun Pharma, TARGET PharmaSolutions, TIMBER, UCB and Zura Bio.

## ETHICAL APPROVAL

The trial protocol was approved by the following institutional review boards or independent ethics committees: Iniciativa y Reflexión Bioética (Argentina), Veritas IRB Inc. (Canada), Health Research Ethics Board of Alberta Clinical Trials Committee (Canada), Ethik‐Kommission der Universitaet Wuerzburg (Germany), Comité Etico de Investigacion Clínica de Cantabria (Spain), Health Research Authority IRB/EC (United Kingdom), University Hospital Southampton NHS Foundation Trust IRB/EC (United Kingdom), North West—Liverpool Central Research Ethics Committee (United Kingdom), London North West University Healthcare NHS Trust (United Kingdom), Northern Care Alliance NHS Group (United Kingdom), Medical Research Council Ethics Committee for Clinical Pharmacology (Hungary), Seoul National University Hospital IRB (Republic of Korea), Pusan National University Hospital IRB (Republic of Korea), Konkuk University Medical Center IRB (Republic of Korea), Korea University Guro Hospital IRB/IEC (Republic of Korea), Supreme Bioethics Committee (Poland), On Dokuz Mayıs Üniversitesi Tıp Fakültesi Klinik Araştırmalar Etik Kurulu (Turkey), National Taiwan University Hospital Research Ethics Committee (Taiwan), Chang Gung Medical Foundation Institutional Review Board (Taiwan), Sterling Institutional Review Board (United States of America).

## ETHICS STATEMENT

The trial was conducted according to the Declaration of Helsinki, the International Council for Harmonisation of Technical Requirements for Pharmaceuticals for Human Use guidelines for Good Clinical Practice and applicable local regulations. The trial protocol was approved by each site's institutional review board or independent ethics committee. Participants/legally designated representatives provided written informed consent before any study procedures.

## Supporting information


Data S1.

## Data Availability

The data sharing policy of Johnson & Johnson is available at https://innovativemedicine.jnj.com/our‐innovation/clinical‐trials/transparency. As noted on this site, requests for access to the study data can be submitted through the Yale Open Data Access [YODA] Project site at http://yoda.yale.edu.
