# Supplementary material for: Durability of response to icotrokinra for high‐impact site psoriasis: 1‐year ICONIC‐TOTAL findings
Source: J Eur Acad Dermatol Venereol. 2026 May 23;40(9):1502–14. doi: 10.1111/jdv.70471 (PMC13505761; doi:10.1111/jdv.70471)
Supplement: Supplementary file 1 [file JDV-40-1502-s001.docx]

# Title:

Durability of Response to Icotrokinra for High-Impact Site Psoriasis: 1-Year ICONIC-TOTAL Findings

**Authors**:

Richard B. Warren, BSc, MBChB, MRCP PhD, FRCP;^1^ Melinda Gooderham, MD, MSc FRCPC;^2^ Edward Lain, MD, MBA, FAAD;^3^ Robert Bissonnette, MD;^4^ Yu-Huei Huang, MD;^5^ Julien Ringuet, MD;^6^ Matthias Hoffmann, MD;^7^ Andreas Pinter, MD, PhD;^8^ Joseph F. Merola, MD, MMSc;^9^ Jessica H. Rubens, MD;^10^ Dorothy Fan, PharmD;^10^ Ming-Chun Hsu, PhD;^11^ Shu Li, PhD;^10^ Ya-Wen Yang, MD;^12^ Cynthia M. C. DeKlotz, MD;^10^ Eingun James Song, MD^13^

**Affiliations**:

1. Dermatology Centre, Northern Care Alliance NHS Foundation Trust & Division of Musculoskeletal and Dermatological Sciences, Manchester NIHR Biomedical Research Centre, Manchester Academic Health Science Centre, University of Manchester, United Kingdom
2. SKiN Centre for Dermatology, Department of Medicine at Queen’s University and Probity Medical Research, Peterborough, Ontario, Canada
3. Austin Institute for Clinical Research, Sanova Dermatology, Austin, TX, USA
4. Innovaderm Research, Montreal, Quebec, Canada
5. Department of Dermatology, Chang Gung Memorial Hospital and College of Medicine, Chang Gung University, Taoyuan City, Taiwan
6. Centre de Recherche Dermatologique du Québec Métropolitain, Québec and Department of Dermatology, McGill University, Montreal, Quebec, Canada
7. Dermatology Practice Dr. Matthias Hoffmann, Witten, Germany
8. University Hospital Frankfurt am Main, Frankfurt am Main, Germany
9. Department of Dermatology and Department of Medicine, Division of Rheumatology, UT Southwestern Medical Center and O'Donnell School of Public Health, Dallas, TX, USA
10. Johnson & Johnson, Spring House, PA, USA
11. Johnson & Johnson, Wayne, PA, USA
12. Johnson & Johnson, Horsham, PA, USA
13. Frontier Dermatology, Mill Creek, WA, USA

**Corresponding author:**

Richard B. Warren, MD, PhD

Dermatology Centre, Northern Care Alliance NHS Foundation Trust & Division of Musculoskeletal and Dermatological Sciences, Manchester NIHR Biomedical Research Centre, Manchester Academic Health Science Centre, University of Manchester, United Kingdom

E-mail: Richard.Warren@manchester.ac.uk

Phone: +44 (0)161 206 4344

# Supplemental materials

## Supplemental methods

### Outcomes and analyses

Reported here are rates of achievement of the following endpoints through W52: IGA 0/1 and a ≥2-grade improvement from baseline; IGA 0; CMI in PSSD Itch score among participants with baseline PSSD Itch score ≥4; DLQI 0/1 among participants with baseline DLQI >1; ss-IGA 0/1, ss-IGA 0, and PSSI improvement of ≥90% (PSSI 90) among participants with baseline ss-IGA score ≥3; CMI in the Scalp Itch NRS score among participants with baseline Scalp Itch NRS ≥4 and ss-IGA score ≥3; sPGA-G 0/1 and sPGA-G 0 among participants with baseline sPGA-G score ≥3; CMI in GPSS Genital Itch NRS among participants with baseline GPSS Genital Itch NRS ≥4 and sPGA-G score ≥3; GenPs-SFQ Item 2 score of 0/1 and GenPs-SFQ Item 2 score of 0 among participants with baseline GenPs-SFQ Item 2 score ≥2 and sPGA-G score ≥3; hf-PGA 0/1 and hf-PGA 0 among participants with baseline hf-PGA score ≥3; and mNAPSI score of 0, among participants with baseline mNAPSI score >0. Percent change from baseline through W52 in mNAPSI score among participants with baseline mNAPSI score >0 was also evaluated.

Safety was evaluated in participants who received ≥1 dose of study drug. Safety assessments included frequency and type of AEs summarized by treatment group. To account for differing follow-up periods across treatment groups, the number of participants with ≥1 type of event per 100 patient-years (PY) was also reported, with associated 95% CIs based on the Wald statistic using the normal assumption for AEs, SAEs, infections, and gastrointestinal AEs; an exact method assuming the observed number of events follows a Poisson distribution was used for serious infections, AEs leading to discontinuation, and malignancies.

Nonresponder imputation was used to handle missing data. Participants who discontinued the study drug due to lack of efficacy, an AE of worsening psoriasis, or initiation of a protocol-prohibited medication or therapy during the study that could improve psoriasis prior to W52 were considered as nonresponders after discontinuation. For continuous endpoints, nonresponders were considered to have a zero change from baseline after the event. In the event of discontinuation of the study drug prior to W52 for reasons other than those outlined above, observed data were used. For binary outcomes, participants with missing data after the above data handling rules were considered as nonresponders. For the placebo group, only participants who crossed over to receive icotrokinra were included in the analyses after Week 16.

## Event narrative

A 72-year-old female participant with an adverse event of malignant melanoma had a history of melanoma beginning in 2021 and dysplastic nevi. Assessment for eligibility occurred on December 13, 2023. The participant was randomized to the icotrokinra treatment arm and received the first dose on January 22, 2024. During the investigational period, the participant had an adverse event of left posterior lateral distal thigh melanoma in situ and underwent wide excision. The study sponsor assessment was that this event was unrelated to the study drug.

A 71-year-old male with a history of neutropenia (since 2019) who was randomized to icotrokinra had a nonserious AE of chronic lymphocytic leukemia. The study sponsor assessment was that this event was unrelated to the study drug based on the participant’s age and a confounding history of neutropenia.

## Supplemental Figures

## Figure S1. GenPs-SFQ Item 2 score of 0 through Week 52 among participants with genital psoriasis

GenPs-SFQ item 2 evaluated among participants with baseline score ≥2 and sPGA-G ≥3. GenPs-SFQ Item 2 (In the past week, how often did your genital psoriasis limit the frequency of your sexual activity?) range: 0 (never), 1 (rarely), 2 (sometimes), 3 (often), 4 (always).

CI: confidence interval; GenPs-SFQ: Genital Psoriasis Sexual Frequency Questionnaire; sPGA-G: Static Physician’s Global Assessment of Genitalia.

## Figure S2. Proportion of participants achieving (A) hf-PGA 0/1 and (B) hf-PGA 0 among participants with hand/foot psoriasis; and (C) percent change from baseline in mNAPSI, among participants with nail psoriasis, through Week 52

hf-PGA evaluated among participants with baseline score ≥3. mNAPSI evaluated among participants with baseline score >0. hf-PGA range: 0 (clear), 1 (almost clear), 2 (mild), 3 (moderate), 4 (severe). mNPASI range: 0-130; higher scores indicate worse severity.

CI: confidence interval; hf-PGA: Physician's Global Assessment of the hands and/or feet; mNAPSI: modified nail psoriasis severity index.

**A**

**B**

**C**
